# Risk factors for venous bleeding complication at the femoral puncture site after catheter ablation of atrial fibrillation

**DOI:** 10.1002/joa3.12378

**Published:** 2020-06-01

**Authors:** Hirofumi Arai, Akira Mizukami, Yoshihiro Hanyu, Takuya Kawakami, Yuki Shimizu, Jiro Hiroki, Kenji Yoshioka, Hirofumi Otani, Shunsuke Kuroda, Ryota Iwatsuka, Daisuke Ueshima, Tatsuya Hayashi, Akihiko Matsumura, Masahiko Goya, Tetsuo Sasano

**Affiliations:** ^1^ Department of Cardiology Kameda Medical Center Kamogawa Japan; ^2^ Department of Cardiovascular Medicine Tokyo Medical and Dental University Bunkyo, Tokyo Japan

**Keywords:** atrial fibrillation, body mass index, catheter ablation, femoral vein, hemostasis

## Abstract

**Background:**

Venous bleeding complication is often observed after catheter ablation of atrial fibrillation (AF), but the risk factors remain unclear.

**Methods:**

We retrospectively evaluated 570 consecutive patients who underwent catheter ablation of AF from April 2012 to March 2017. After the procedure, the sheaths were removed, and hemostasis was obtained by manual compression followed by application of rolled gauze with elastic bandage and continuous pressure to the puncture site. We evaluated the risk factors for venous bleeding complications defined as hemorrhage from the puncture site that needed recompression after removal of the elastic bandage and rolled gauze.

**Results:**

After excluding 11 patients because of missing data, 559 patients (395 [70.7%] men, mean age: 65.6 ± 8.7 years) were included for analysis. Venous bleeding complication was observed in 213 patients (38.1%). In the multivariate logistic regression analysis, low body mass index (BMI; odds ratio [OR] 0.95, 95% CI 0.90‐1.00, *P* = .04), short compression time (OR 0.77, 95% CI 0.68‐0.88, *P* < .001), and antiplatelet therapy (OR 1.86, 95% CI 1.09‐3.16, *P* = .02) were independent risk factors for venous bleeding complication.

**Conclusions:**

Low BMI, short compression time, and antiplatelet therapy were independent risk factors for venous bleeding complication after catheter ablation of AF. Longer compression time may be needed for patients with low BMI and/or those receiving antiplatelet therapy.

## Graphical abstract

We retrospectively evaluated 570 consecutive patients who underwent catheter ablation of atrial fibrillation. The risk factors for venous bleeding complications were analyzed. Low BMI, short compression time, and antiplatelet therapy were found to be the significant risk factors.

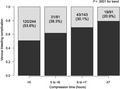

## 1 INTRODUCTION

Atrial fibrillation (AF) is a common arrhythmia, and its prevalence and incidence has increased during the past 50 years.^1^ Haïssaguerre et al reported in 1998 that AF was mainly triggered by ectopic beats originating from pulmonary veins,^2^ and pulmonary vein isolation (PVI) has been the core of catheter ablation of AF.

Radiofrequency (RF) ablation has been commonly used. However, cryo‐balloon ablation was shown to be as effective as RF ablation for paroxysmal AF with shorter procedure time^3^ and is gaining popularity. Both procedures need 2 or 3 large‐sized sheaths from the femoral vein for insertion of the ablation catheter and mapping catheter and intracardic echocardiography. Vascular complications often occur at the puncture site after PVI. The reported bleeding complication rate is 0%‐29% and accounts for most complications of PVI.^4^, ^5^, ^6^, ^7^, ^8^ Even though venous bleeding complication is rarely fatal and may be less important than other major complications, the frequency is relatively high. It significantly influences the patient's quality of life with fear and discomfort from bleeding and prolonged restriction of movement and can lead to hemorrhagic shock if left untreated. Even though the risks of atrial puncture site complication after coronary interventions have been discussed, the risk factors of venous bleeding complications after catheter ablation are still unknown.

In cases of femoral artery puncture, obesity is known as a risk factor for puncture site complications.^9^, ^10^ However, the risk of venous bleeding complication and correlation with BMI are unclear. Obesity is known as a risk factor for venous thromboembolism (VTE).^11^ Coagulation and platelet activation, endothelial cell activation, blood flow reduction, and local compression of vein were some of the factors that may lead to VTE in patients with obesity.^12^ Conversely, we hypothesized that low body mass index (BMI) was a risk factor for venous bleeding complication after PVI because of the lack of these effects of obesity and on the basis of our clinical experience.

## 2 METHODS

### 2.1 Study design

We retrospectively evaluated 570 consecutive patients who underwent PVI between April 2012 and March 2017 at Kameda Medical Center. We decided the indication of PVI according to the 2011 Guidelines for Non‐Pharmacotherapy of Cardiac Arrhythmias of the Japanese Circulation Society,^13^ which is consistent with the American College of Cardiology/American Heart Association/Heart Rhythm Society and European Society of Cardiology/European Heart Rhythm Association guidelines. Data on baseline characteristics at the time of catheter ablation, procedure characteristics, and postprocedure care regarding hemostasis of the puncture site were collected. We excluded cases with missing data on any of these factors. The outcome assessed was venous bleeding complication that needed recompression after bandage release, and the contributing factors for this end point were analyzed.

### 2.2 Peri‐procedural anticoagulation control

Warfarin therapy, with PT‐INR controlled between 2.0 and 3.0 for patients aged <70 years and between 1.6 and 2.6 for patients aged >70 years according to the recommendation of the Guidelines for Drug Treatment of Arrhythmia of Japanese Circulation Society,^14^ was continued. The morning dose was skipped in twice‐per‐day prescriptions of direct oral anticoagulants, including apixaban and dabigatran. However, dabigatran was not skipped after the introduction of idarcizumab in November 2016. In the cases of once‐per‐day prescriptions, including rivaroxaban and edoxaban, they were taken in the evening and continued. Intravenous injection of unfractionated heparin was used to maintain an activated clotting time of >300 seconds during the procedure. The recommended protamine sulfate dose was injected before the sheath removal after the procedure. The day after the procedure, anticoagulation drugs were taken as usual.

### 2.3 Sheath introduction during catheter ablation

All the patients were treated with RF ablation or cryo‐balloon ablation. Mainly, three sheaths were introduced from the femoral vein for ablation, mapping, and insertion of ultrasonography catheters. We used an 8.5‐Fr steerable sheath, Agilis™ NxT (Abbott) or Destino™ (Oscor) for RF ablation and a 12‐Fr steerable sheath, FlexCath Advance™ (Medtronic), for cryo‐balloon ablation. The Swartz™ Braided Transseptal Guiding Introducer SL1 8.5 F (Abbott) was used for the mapping catheter. The Radifocus Introducer IIH® 9‐ or 10‐Fr sheath (Terumo) was used for intracardiac echocardiography. The puncture site was decided on the discretion of the operator, but we mainly inserted the sheaths for ablation and mapping catheter from the right femoral vein and the sheath for the ultrasonography catheter from the left femoral vein from April 2012 to March 2015. Thereafter, we inserted the three sheaths only from the right femoral vein.

We monitored the arterial blood pressure in all the cases with a 20‐G monitoring catheter or the Radifocus Introducer IIH^®^ 4‐Fr sheath (Terumo) for the patients who received concurrent coronary angiography. We inserted these catheters from the right femoral artery.

### 2.4 Hemostasis after the procedure

**Figure 1:**
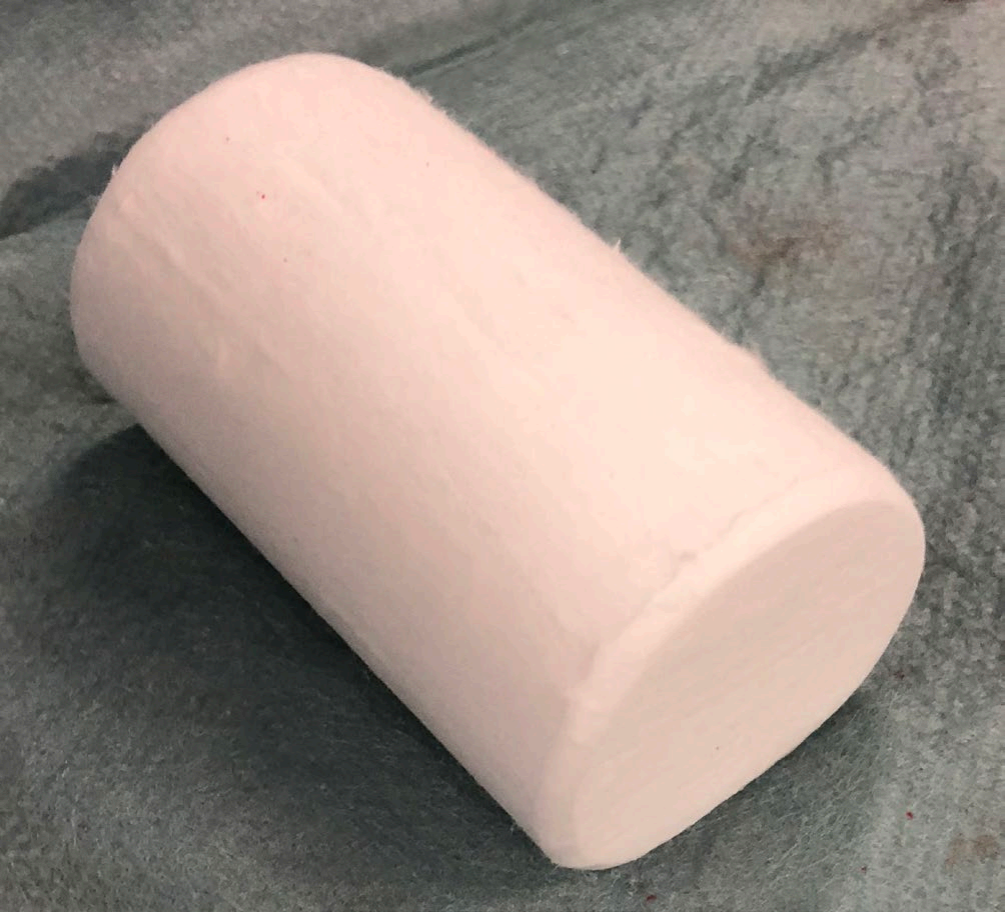
Angio‐hemostasis roll. We used an angio‐hemostasis roll for all the patients to compress the puncture site after manual compression

**Figure 2:**
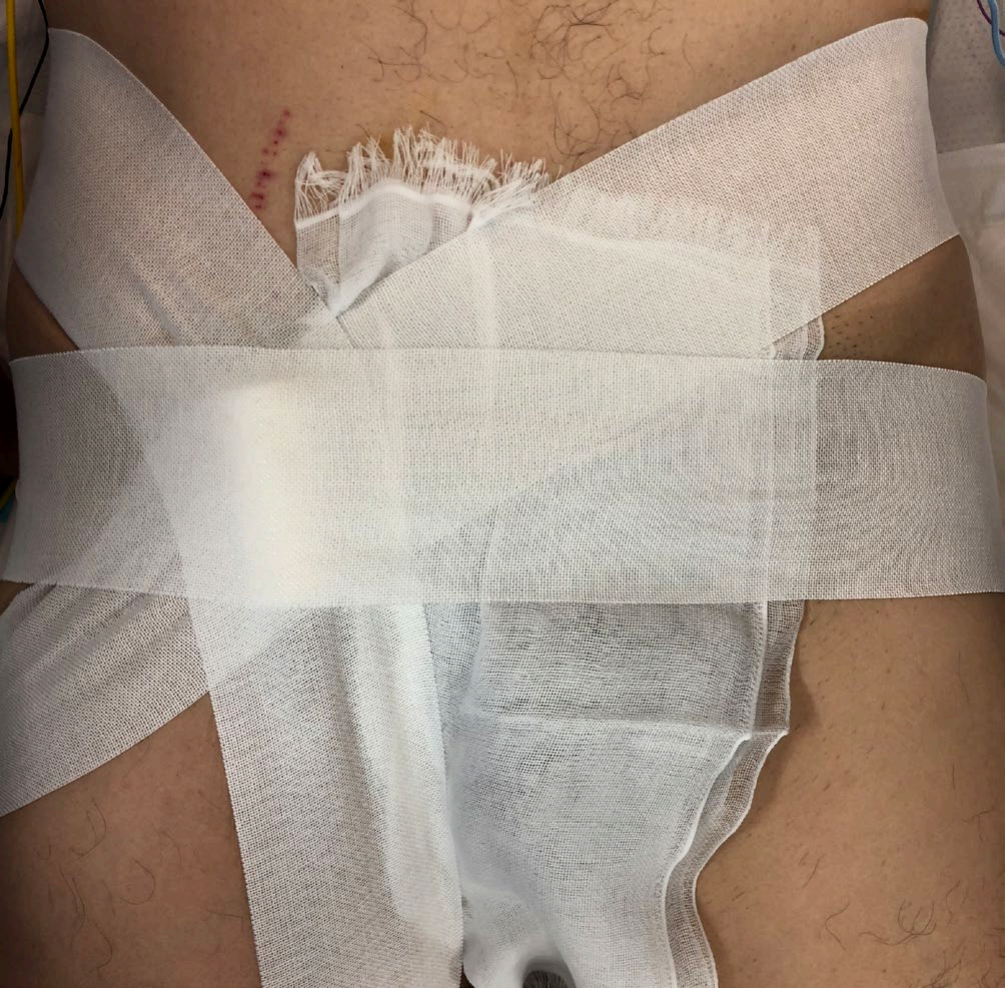
Angio‐hemostasis roll with an elastic bandage. An angio‐hemostasis roll was fixed with an elastic bandage. We instructed the patients not to move their hip joint on the puncture side until the bandage was removed

We applied manual compression after sheath removal until hemostasis was achieved. Thereafter, a 30‐ × 60‐mm angio‐hemostasis roll (Hakujuji; Figure 1) was used with an elastic bandage to apply continuous pressure (Figure 2). Continuous pressure was applied for 4 hours until November 2015, when we prolonged the time to 6 hours owing to significant episodes of venous bleeding complications. We did not use figure‐of‐eight sutures, purse string sutures, and any other specific devices for hemostasis in this population. Compression time was dispersed because all the patients were checked for hemostasis by the medical staff at the end of the continuous pressure application, and the availability of the medical staff varied. Additional compression was applied by the physician's discretion if rehemorrhage occurred from the puncture site.

### 2.5 Statistical analyses

The unpaired t test and Mann‐Whitney *U* test were used for continuous variables with normal and nonnormal distributions, respectively, and the Fisher exact test was used for categorical variables to compare data between the rehemorrhage and nonhemorrhage groups. The continuous variables are described as mean ± SD for those with normal distribution and median with interquartile range for those with nonnormal distribution. Categorical variables are described as n (%). We calculated odds ratios (OR), 95% confidence intervals (CI), and p values with logistic regression analysis. We used all the parameters that we gathered for univariate logistic regression analysis. We selected the factors with *P* < .1 in the univariate logistic regression analysis for multivariate logistic regression analysis to identify risks of venous bleeding complication. The Cochran‐Armitage trend test was applied to compare the rate of venous bleeding complication according to compression time. We considered *P* < .05 as statistically significant. We analyzed all data with EZR version 1.36 (Saitama Medical Center, Jichi Medical University, Saitama, Japan), which is a graphical user interface for the R software (The R Foundation for Statistical Computing).^15^

## 3 RESULTS

Five hundred and seventy patients were included, and 11 patients (1.9%) with missing data were excluded; thus, 559 patients were evaluated. The patients’ mean age was 65.6 ± 8.7 years, and 395 patients (70.7%) were male. The number of patients who had venous bleeding complication was 213 (38.1%). All the patients had minimal bleeding, and none had minor and major bleeding, as defined by the TIMI bleeding criteria.^16^ No deep vein thrombotic events were found during admission. No significant differences were found between the two groups with the exception of BMI, compression time, cryo‐ablation, antiplatelet therapy, apixaban, and digoxin therapies (Table 1). In the univariate logistic regression analysis, cryo‐ablation (OR 0.422, 95% CI 0.205‐0.868, *P* = .019), compression time (OR 0.738, 95% CI 0.653‐0.833, *P* < .001), apixaban therapy (OR 0.618, 95% CI 0.393‐0.972, *P* = .037), digoxin therapy (OR 2.97, 95% CI 1.23‐7.21, *P* = .016), and antiplatelet therapy (OR 1.92, 95% CI 1.16‐3.19, *P* = .012) were significant predictors of venous bleeding complication (Table 2). However, in the multivariate logistic regression analysis, low BMI (OR 0.948, 95% CI 0.9‐0.998, *P* = .042), short compression time (OR 0.774, 95% CI 0.679‐0.882, *P* < .001), and antiplatelet therapy (OR 1.86, 95% CI 1.09‐3.16, *P* = .022) were the only significant independent risk factors for venous bleeding complication (Table 3).

**Table 1:** Baseline characteristics

	All	Hemorrhage (−)	Haemorrhage (+)	*P* value
n	559	346	213	
Male, n (%)	395 (70.7)	236 (68.2)	159 (74.6)	.13
Age, y	65.6 ± 8.69	65.4 ± 8.88	65.9 ± 8.39	.5
BMI (kg/m^2^)	24.4 ± 3.62	24.6 ± 3.73	24 ± 3.42	.05
CHA2DS2‐VASc	2 (1‐3)	2 (1‐3)	2 (1‐3)	.54
CHADS2	1 (0‐2)	1 (0‐2)	1 (1‐2)	.45
HAS‐BLED	1 (1‐2)	1 (1‐2)	2 (1‐2)	.34
LVEF (%)	66 (61‐71)	66 (61‐70)	66 (61‐71)	.38
BNP (pg/ml)	165.6 ± 305.7	166.2 ± 270	164.8 ± 356.9	.96
eGFR, ml/(min·1.73 m^2^)	57.3 (47.9‐61.4)	57.9 (48.2‐61.2)	57.8 (47.8‐61.4)	.84
Dialysis, n (%)	15 (2.7)	9 (2.6)	6 (2.8)	1
Diagnosis, n (%)				
PAF	318 (56.9)	195 (56.4)	123 (57.7)	.79
PEF	236 (42.2)	146 (42.2)	90 (42.3)	1
AFL	121 (21.6)	77 (22.2)	44 (20.7)	.67
	65 (11.6)	43 (12.4)	22 (10.3)	.5
Procedures, n (%)				
Cryo	47 (8.4)	37 (10.7)	10 (4.7)	.02
Procedure time (h)	3.82 ± 1.15	3.82 ± 1.17	3.82 ± 1.12	.94
Compression time (h)	5.8 ± 1.89	6.11 ± 2.07	5.28 ± 1.38	<.001
Past history, n (%)				
Hypertension	295 (52.8)	178 (51.4)	117 (54.9)	.43
Dyslipidemia	176 (31.5)	116 (33.5)	60 (28.2)	.19
Diabetes mellitus	97 (17.4)	63 (18.2)	34 (16)	.57
Heart failure	127 (22.7)	86 (24.9)	41 (19.2)	.15
Stroke	51 (9.1)	36 (10.4)	15 (7)	.23
Vascular disease	76 (13.6)	44 (12.7)	32 (15)	.45
Anticoagulation, n (%)				
NOAC	376 (67.3)	241 (69.7)	135 (63.4)	.14
Apixaban	109 (19.5)	77 (22.3)	32 (15.0)	.04
Dabigatran	88 (15.7)	47 (13.6)	41 (19.2)	.09
Edoxaban	16 (2.9)	13 (3.8)	3 (1.4)	.12
Rivaroxaban	163 (29.2)	104 (30.1)	59 (27.7)	.57
Warfarin	183 (32.7)	105 (30.3)	78 (36.6)	.14
Antiarrhythmic medications, n (%)				
Disopyramide	4 (0.1)	3 (0.1)	2 (0.1)	1
Cibenzoline	66 (11.8)	35 (10.1)	31 (14.6)	.14
Aprindine	36 (6.4)	21 (6.1)	15 (7)	.72
Flecainide	73 (13.1)	47 (13.6)	26 (12.2)	.7
Pilsicainide	106 (19)	67 (19.4)	39 (18.3)	.82
Propafenone	21 (3.8)	10 (3)	11 (5.2)	.18
Amiodarone/Sotalol	76 (13.6)	48 (13.9)	28 (13.1)	.9
Bepridil	113 (20.2)	64 (18.5)	49 (23)	.23
NondihydropyridineCCB	100 (17.9)	61 (17.6)	39 (18.3)	.91
ACE‐I/ARB	218 (39)	133 (38.4)	85 (39.9)	.79
β blocker	266 (47.6)	159 (46)	107 (50.2)	.34
Antiplatelet therapy	69 (12.3)	33 (9.5)	36 (16.9)	.01
SAPT	65 (11.6)	31 (9)	34 (16)	.01
DAPT	4 (0.7)	2 (0.6)	2 (0.9)	.64

**Table 2:** Univariate logistic regression analysis

	OR	95% CI	*P* value
Male	1.37	0.936‐2.01	.11
Age (y)	1.01	0.987‐1.03	.5
BMI (kg/m^2^)	0.954	0.909‐1.0	.053
CHA2DS2‐VASc	0.946	0.844‐1.06	.23
CHADS2	0.906	0.771‐1.06	.23
HAS‐BLED	1.06	0.898‐1.25	.5
LVEF (%)	1.01	0.994‐1.03	.23
BNP (pg/ml)	1	0.999‐1.0	.96
eGFR, ml/(min·1.73 m^2^)	1	0.982‐1.02	.85
Dialysis	1.09	0.381‐3.09	.88
Cryo‐balloon	0.422	0.205‐0.868	.019
Procedure time (h)	1.01	0.87‐1.17	.94
Compression time (h)	0.738	0.653‐0.833	<.001
Hypertension	1.15	0.817‐1.62	.42
Dyslipidemia	0.778	0.536‐1.13	.19
Diabetes mellitus	0.853	0.54‐1.35	.5
Heart failure	0.721	0.474‐1.1	.13
Stroke	0.652	0.348‐1.22	.18
Vascular disease	1.21	0.74‐1.98	.44
NOAC	0.754	0.526‐1.08	.13
Apixaban	0.618	0.393‐0.972	.037
Dabigatran	1.52	0.958‐2.4	.075
Edoxaban	0.366	0.103‐1.3	.12
Rivaroxaban	0.891	0.611‐1.3	.55
Warfarin	1.33	0.924‐1.9	.13
Disopyramide	1.08	0.18‐6.54	.93
Cibenzoline	1.51	0.903‐2.54	.12
Aprindine	1.17	0.591‐2.33	.65
Flecainide	0.885	0.53‐1.48	.64
Pilsicainide	0.933	0.602‐1.45	.76
Propafenone	1.83	0.764‐4.38	.18
Amiodarone/sotalol	0.94	0.569‐1.55	.81
Bepridil	1.32	0.866‐2	.2
Nondihydropyridine CCB	1.05	0.672‐1.63	.84
Digoxin	2.97	1.23‐7.21	.016
ACE‐I/ARB	1.06	0.75‐1.51	.73
β‐Blocker	1.19	0.843‐1.67	.33
Antiplatelet therapy	1.92	1.16‐3.19	.012

Abbreviations: ACE‐I, angiotensin‐converting enzyme inhibitor; AFL, atrial flutter; ARB, angiotensin receptor blocker; AT, atrial tachycardia; CCB, calcium channel blocker; LVEF, left ventricular ejection fraction; NOAC, novel oral anticoagulants; PAF, paroxysmal atrial fibrillation; PEF, persistent atrial fibrillation.

**Table 3:** Multivariate logistic regression analysis

	OR	95% CI	*P* value
BMI (kg/m^2^)	0.948	0.9‐0.998	.042
Cryo‐balloon	0.819	0.375‐1.79	.62
Compression time (h)	0.774	0.679‐0.882	<.001
Apixaban therapy	0.858	0.526‐1.4	.54
Dabigatran therapy	1.32	0.805‐2.16	.27
Digoxin therapy	1.91	0.763‐4.8	.17
Antiplatelet therapy	1.86	1.09‐3.16	.022

**Figure 3:**
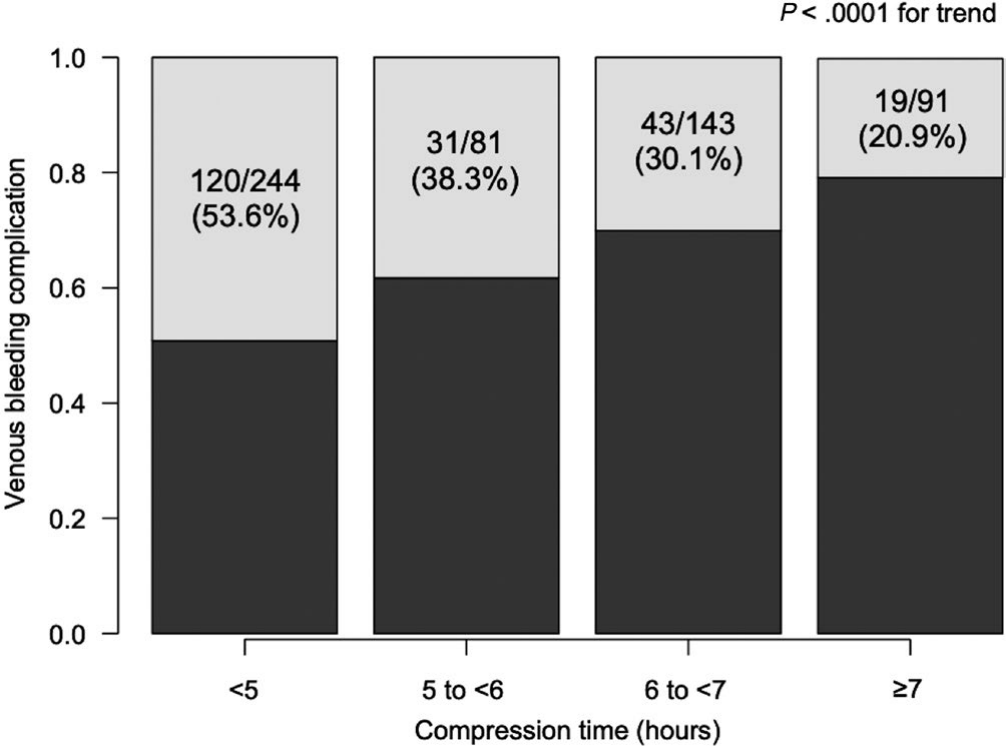
Venous bleeding complication rate in the compression time groups. The white bars indicate the incidence rate of venous bleeding complication. As the compression time was prolonged, the incidence rate of venous bleeding complication significantly decreased (*P* < .0001)

We divided the patients into four groups according to compression time (<5 hours, 5 to <6 hours, 6 to <7 hours, and ≥7 hours) and evaluated the incidence of venous bleeding complication according to the groups. The incidence of venous bleeding complication significantly decreased as the compression time became longer, with a bleeding rate of 53.6% with <5 hours to 20.9% with ≥7 hours (*P* < .0001 for trend; Figure 3).

## 4 DISCUSSION

This study retrospectively investigated the risk factors for venous bleeding complication after PVI. The results showed that low BMI, short compression time, and antiplatelet therapy were independent predictors of this end point.

Many reports have described complications after PVI, and bleeding of the puncture site is one of the most frequent complications. However, to the best of our knowledge, no studies have investigated the risk factors for venous bleeding complication. Venous bleeding complication prolongs restriction of movement, and uncontrollable hemorrhage may need discontinuation of anticoagulation, which will lead to an increase in the risk of thromboembolism. From this point of view, control of venous bleeding complication is important, and knowledge of the risk factors for venous bleeding complication is crucial.

The value of BMI had little differences between patients with and without bleeding. However, the differences reached statistical significance, and BMI has been clearly shown to be a significant predictor in multivariate analysis. A high number of study subjects may have contributed to the detection of the difference, and differences in many other risk factors of venous bleeding complications between the two groups may also have contributed.

Obesity is known as a risk factor for VTE.^11^ Coagulation and platelet activation, endothelial cell activation, blood flow reduction, and local compression of vein are some of the proposed mechanisms.^12^ Lack of these effects of obesity may have resulted in the higher incidence of venous bleeding complication in patients with low BMI.

In the previous reports, anticoagulation was continued in most cases before AF ablation,^7^ and the compression time was 4 hours in all the cases.^8^ Nevertheless, the incidence of venous bleeding complication in this study was higher than that in the previous reports. The BMI in our cohort was lower than that in the previously reported cohort. This may be one of the reasons why the incidence rate of venous bleeding complication in our study was higher than the reported rate. The odds ratio of BMI for venous bleeding complication was 0.948 for 1 BMI increase, which corresponds to 0.766 for 5 BMI increases and 0.586 for 10 BMI increases.

Several procedures or devices such as the figure‐of‐eight suture,^17^, ^18^ purse string suture,^8^ Perclose device,^19^ and Angio‐Seal device^20^ were reported to be useful for venous hemorrhage. Among these procedures, the use of the figure‐of‐eight suture is low‐cost, easy, and effective. They may play an important role in reducing the incidence rate of the venous bleeding complications.

Compression time was also a significant factor for venous bleeding complication. Patients who undergo PVI are anticoagulated, and the time required for venous hemostasis may be longer than that required for other procedures. Enough time for hemostasis is important; however, longer compression extends the patient's restriction time and may increase the risk of venous thrombosis. Procedures to help hemostasis, such as figure‐of‐eight suture, may contribute to decreased compression time and improve patient satisfaction.

This study has several limitations. First, this is a retrospective observational study performed in a single center in Japan, and the results may not be generalized. The venous bleeding complication in this study was defined as bleeding that needed recompression, which was decided according to the caring physician's judgment. Even though the judgment is clear in most cases, it might be deferred in cases of mild oozed bleeding. Even though the methods of venous puncture and compression were unified, differences among operators may have existed. The size of the sheath used, the puncture site, and the compression time differed according to the patient and time. Unmeasured factors may influence the risk of venous bleeding complication. The number of patients receiving dual antiplatelet therapy was small, and the difference in bleeding risk between single or dual antiplatelet therapy could not be analyzed. The use of vascular ultrasound during venous puncture was at discretion of the operator, and was not used routinely, which may have influenced the risk of puncture site complication.

## 5 CONCLUSIONS

Low BMI, short compression time, and antiplatelet therapy were the significant risk factors for venous bleeding complication after PVI. The use of hemostatic methods such as figure‐of‐eight suture should be considered especially for patients with these risk factors.

## CONFLICT OF INTEREST

There is no conflict of interest.

## References

[1] Schnabel RB , Yin X , Gona P , Larson MG , Beiser AS , McManus DD , et al. 50 year trends in atrial fibrillation prevalence, incidence, risk factors, and mortality in the Framingham Heart Study: A cohort study. Lancet. 2015;386(9989):154–62. 25960110 10.1016/S0140-6736(14)61774-8 PMC4553037

[2] Haïssaguerre M , Jaïs P , Shah DC , Takahashi A , Hocini M , Quiniou G , et al. Spontaneous initiation of atrial fibrillation by ectopic beats originating in the pulmonary veins. N Engl J Med. 1998;339(10):659–66. 9725923 10.1056/NEJM199809033391003

[3] Kuck K‐H , Brugada J , Fürnkranz A , Metzner A , Ouyang F , Chun KRJ , et al. Cryoballoon or radiofrequency ablation for paroxysmal atrial fibrillation. N Engl J Med. 2016;374(23):2235–45. 27042964 10.1056/NEJMoa1602014

[4] Deshmukh A , Patel NJ , Pant S , Shah N , Chothani A , Mehta K , et al. In‐hospital complications associated with catheter ablation of atrial fibrillation in the United States between 2000 and 2010: Analysis of 93 801 procedures. Circulation. 2013;128(19):2104–12. 24061087 10.1161/CIRCULATIONAHA.113.003862

[5] Shah RU , Freeman JV , Shilane D , Wang PJ , Go AS , Hlatky MA . Procedural complications, rehospitalizations, and repeat procedures after catheter ablation for atrial fibrillation. J Am Coll Cardiol. 2012;59(2):143–9. 22222078 10.1016/j.jacc.2011.08.068 PMC5340189

[6] Hosseini SM , Rozen G , Saleh A , Vaid J , Biton Y , Moazzami K , et al. Catheter ablation for cardiac arrhythmias: utilization and in‐hospital complications, 2000 to 2013. JACC Clin Electrophysiol. 2017;3(11):1240–8. 29759619 10.1016/j.jacep.2017.05.005

[7] Muthalaly RG , John RM , Schaeffer B , Tanigawa S , Nakamura T , Kapur S , et al. Temporal trends in safety and complication rates of catheter ablation for atrial fibrillation. J Cardiovasc Electrophysiol. 2018;29(6):854–60. 29570900 10.1111/jce.13484

[8] Jackson N , McGee M , Ahmed W , Davies A , Leitch J , Mills M , et al. Groin haemostasis with a purse string suture for patients following catheter ablation procedures (GITAR Study). Heart Lung Circ. 2019;28(5):777–83. 29685719 10.1016/j.hlc.2018.03.011

[9] Berry C , Kelly J , Cobbe SM , Eteiba H . Comparison of femoral bleeding complications after coronary angiography versus percutaneous coronary intervention. Am J Cardiol. 2004;94(3):361–3. 15276106 10.1016/j.amjcard.2004.04.036

[10] Waksman R , King SB , Douglas JS , Shen Y , Ewing H , Mueller L , et al. Predictors of groin complications after balloon and new‐device coronary intervention. Am J Cardiol. 1995;75(14):886–9. 7732995 10.1016/s0002-9149(99)80681-x

[11] Holst AG , Jensen G , Prescott E . Risk factors for venous thromboembolism: Results from the Copenhagen city heart study. Circulation. 2010;121(17):1896–903. 20404252 10.1161/CIRCULATIONAHA.109.921460

[12] Hunt BJ . The effect of BMI on haemostasis: Implications for thrombosis in women’s health. Thromb Res. 2017;151(Suppl.1):S53–S55. 28262235 10.1016/S0049-3848(17)30068-3

[13] JCS Joint Working Group . Guidelines for non‐pharmacotherapy of cardiac arrhythmias (JCS 2011). Circ J. 2013;77(1):249–274. 23165786 10.1253/circj.cj-66-0054

[14] JCS Joint Working Group . Guidelines for pharmacotherapy of atrial fibrillation (JCS 2013). Circ J. 2014;78(8):1997–2021. 24965079 10.1253/circj.cj-66-0092

[15] Kanda Y . Investigation of the freely available easy‐to‐use software “EZR” for medical statistics. Bone Marrow Transplant. 2013;48(3):452–458. 23208313 10.1038/bmt.2012.244 PMC3590441

[16] Mehran R , Rao SV , Bhatt DL , Gibson CM , Caixeta A , Eikelboom J , et al. Standardized bleeding definitions for cardiovascular clinical trials. Circulation. 2011;123(23):2736–2747. 21670242 10.1161/CIRCULATIONAHA.110.009449

[17] Traullé S , Kubala M , Doucy A , Quenum S , Hermida JS . Feasibility and safety of temporary subcutaneous venous figure‐of‐eight suture to achieve haemostasis after ablation of atrial fibrillation. Europace. 2016;18(6):815–819. 26467404 10.1093/europace/euv266

[18] Aytemir K , Canpolat U , Yorgun H , Evranos B , Kaya EB , Şahiner ML , et al. Usefulness of “figure‐of‐eight” suture to achieve haemostasis after removal of 15‐French calibre femoral venous sheath in patients undergoing cryoablation. Europace. 2016;18(10):1545–1550. 26705565 10.1093/europace/euv375

[19] Mahadevan VS , Jimeno S , Benson LN , McLaughlin PR , Horlick EM . Pre‐closure of femoral venous access sites used for large‐sized sheath insertion with the Perclose device in adults undergoing cardiac intervention. Heart. 2008;94(5):571–572. 17085529 10.1136/hrt.2006.095935

[20] Coto HA . Closure of the femoral vein puncture site after transcatheter procedures using Angio‐Seal. Catheter Cardiovasc Interv. 2002;55(1):16–19. 11793489 10.1002/ccd.10086

